# Acupuncture for pain and pain-related disability in deep infiltrating endometriosis

**DOI:** 10.3389/fpain.2024.1279312

**Published:** 2024-03-08

**Authors:** Giulia Chiarle, Gianni Allais, Silvia Sinigaglia, Gisella Airola, Sara Rolando, Fabiola Bergandi, Salvatore Micalef, Chiara Benedetto

**Affiliations:** ^1^ Women’s Headache Center, Gynaecology and Obstetrics Unit, Sant’Anna Hospital, University of Turin, Turin, Italy; ^2^ Department of Gynaecology and Obstetrics, University of Turin, Turin, Italy; ^3^ Gynaecology and Obstetrics Unit, Sant’Anna Hospital, University of Turin, Turin, Italy

**Keywords:** acupuncture, disability, dysmenorrhea, dyschezia, dyspareunia, endometriosis, pelvic pain

## Abstract

**Objectives:**

To evaluate the efficacy of acupuncture in relieving symptoms (dysmenorrhea, dyspareunia, pelvic pain and dyschezia) intensity, improving functional disability, reducing the number of days per months of dysmenorrhea, the frequency and the efficacy of analgesic use in deep infiltrating endometriosis (DIE). The safety profile was also evaluated.

**Methods:**

The study sample was 34 patients with DIE; for 2 months (T-2, T-1) the women recorded diary notes on the numbers of days of menstruation, the presence, intensity, and disability related to dysmenorrhea, dyspareunia, pelvic pain, and dyschezia. They then received a total of 15 acupuncture treatments over 6 months (T1–T6; once a week for 12 weeks, then once a month for 3 months).

**Results:**

Dysmenorrhea intensity was decreased during treatment. A decrease of at least 50% in number of days of dysmenorrhea, and a decrease in moderate-to-severe disability starting from T1 to T6 was recorded for 58.6% of patients. Dyspareunia intensity steadily decreased starting at T2; the percentage of women with moderate-to-severe disability declined from 73.3% at T-2, to 36.9% at T3, T4, and T5. A decrease in pelvic pain score was noted starting at T1; the percentage of disability decreased from 83.3% at T-2 to 33.3% at T3 and T6. The intensity of dyschezia decreased from T-2 to T3 and T4 and then increased slightly. Analgesic drug use was lower during treatment and its efficacy appeared to be greater.

**Conclusions:**

The limitations notwithstanding our study-findings show that acupuncture was safe and effective in reducing pain intensity and symptoms-related disability. Larger-scale studies are needed to compare acupuncture and pharmacotherapy for endometriosis-related pain.

## Introduction

Endometriosis is a gynecological disorder in which endometrial tissue grows outside the uterus and causes chronic inflammation at the ectopic implant site (1). This benign, estrogen-dependent condition affects between 5% and 15% of women of reproductive age; hallmark symptoms include dysmenorrhea, chronic pelvic pain, dyspareunia, dyschezia, and dysuria (2). Most endometrial implants arise in the pelvic cavity; endometriosis in extrapelvic sites is uncommon. No single classification system adequately classifies endometriosis. The American Society for Reproductive Medicine classification is the most widely used and is useful for physicians to explain the severity of endometriosis in simple terms to the patients. The ENZIAN classification describes deep infiltrating endometriosis (DIE) involving retroperitoneal structures. The revised ENZIAN classification was simplified by dividing retroperitoneal structures into three compartments. The posterior part of the uterus was divided into compartment A consisting of the rectovaginal septum and vagina, compartment B consisting of the uterosacral ligament and pelvic walls, and compartment C consisting of the sigmoid colon and rectum. The severity of the lesion is set to invasiveness <1 cm for grade 1, invasiveness 1–3 cm for grade 2, and invasiveness >3 cm for grade 3 (3). In DIE, lesions extend deeper than 5 mm under the peritoneal surface (4).

The most common strategies to reduce pain symptoms include excision of the endometrial implants (associated with recurrence of pain and need for repeat surgery within 5–7 years after the initial operation in 50%–60% of patients) (5–8); symptomatic pharmacotherapy of questionable efficacy with painkillers and nonsteroidal anti-inflammatory drugs (NSAIDs) (9); hormonal therapy with oral combined contraceptives or progestin alone, gonadotropin-releasing hormone (GnRH) analogues, androgens (danazol), aromatase inhibitors, anti-tumor necrosis factor (TNF) agents, and selective estrogen receptor modulators (SERMs) (10) Despite the wide array of treatment options, pain relief seems elusive and quality of life often unattainable.

Increased attention has been devoted to combining conventional medical treatment with Complementary and Integrative Medicine (CIM). Though not curative, CIM can alter the progression of endometriosis, reduce the number of lesions, relieve symptoms, and lower the relapse rate. Various techniques have been studied in the treatment of endometriosis: Chinese phytotherapy, body and auricular acupuncture, moxibustion, Transcutaneous Electrical Nerve Stimulation, microwave physiotherapy, hypnosis, and thermal biofeedback (11, 12).

Recently, two meta-analysis and reviews showed that acupuncture may be an effective therapy for dysmenorrhea and pelvic pain related to endometriosis (13, 14). The most frequently reported outcome evaluated was dysmenorrhea, while few studies evaluated the others symptoms, quality of life, disability and patient's satisfaction.

The primary endpoint of the present study was a reduction in symptom intensity (dysmenorrhea, dyspareunia, dyschezia, noncyclical pelvic pain) vs. baseline at 3 and 6 months of treatment as measured on a Numerical Rating Scale (NRS) in women previously treated for DIE and receiving one cycle of acupuncture treatment. Secondary endpoints were a reduction in functional disability related to dysmenorrhea, dyspareunia, chronic pelvic pain, and in the number of days per months of dysmenorrhea. Also evaluated were changes in self-reported analgesic use and occurrence of side effects (safety profile of acupuncture treatment).

## Methods

For this pilot study (approved by Ethics Committee A.O.U. Città della Salute e della Scienza of Turin protocol number 0068254), 34 women of reproductive age (mean 33.8 ± 7.3 years, range 19–46) with DIE attending our acupuncture clinic for the treatment of endometriosis-related pain were recruited. Inclusion criteria were: at least one symptoms suggestive of DIE (dysmenorrhea, noncyclical chronic pelvic pain, deep dyspareunia, dyschezia), histological diagnosis of endometriosis confirmed intraoperatively in the 24 months preceding the start of treatment, and clinical/instrumental diagnosis suggestive of persistent or recurrent DIE. Exclusion criteria were: asymptomatic endometriosis, primary dysmenorrhea, other causes of chronic pelvic pain of gynecological or non-gynecological origin, prophylactic treatment during the 6 months prior to the start of the study. Additional exclusion criteria were: physical and mental disorders which in the physicians' opinion could interfere with participation in the study. Participants deemed eligible for entering the study were informed about the method of needle insertion, the sensations they may experience during treatment, transient side effects, the mechanism of action of acupuncture, and the goal of treatment. Written, informed consent was obtained from all participants. The participants received a monthly diary in which they were asked to record for each menstrual period the number of days of menstruation, the presence and intensity of dysmenorrhea and related disability, the presence and intensity of disability related to dyspareunia and pelvic pain, and intensity of dyschezia.

**Figure 1:**
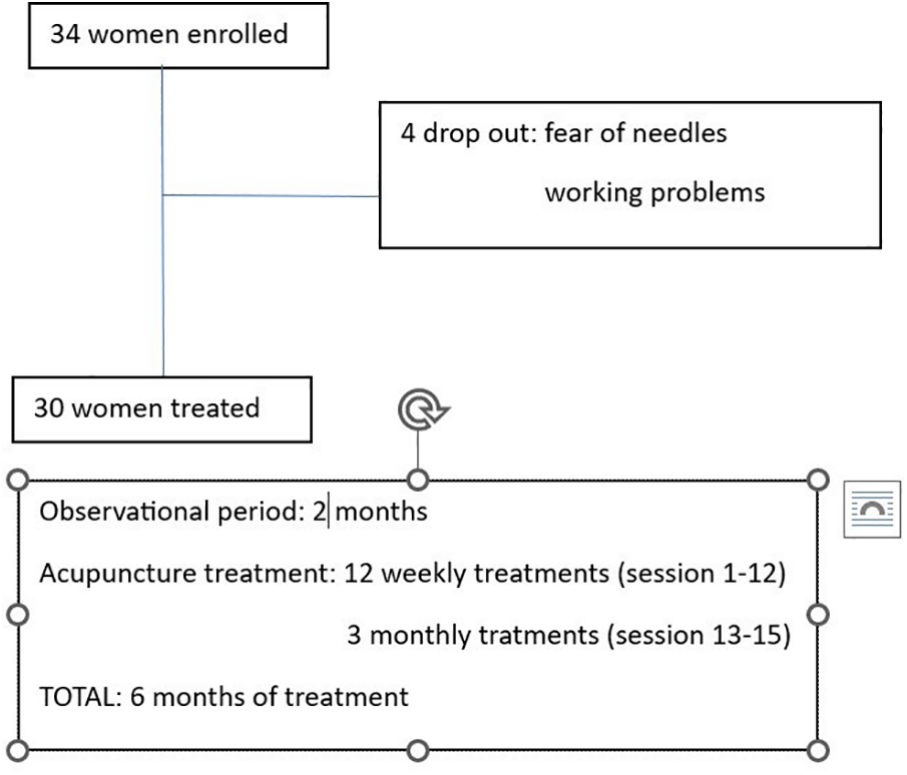
Flow chart of patients enrolled and scheduled treatments.

Symptom intensity was self-rated on a NRS from 0 (no pain) to 10 (worst possible pain); disability related to dysmenorrhea, dyspareunia, and pelvic pain was rated on the Biberoglu and Behrman scale (modified version) (15). Diary entries began on the day of enrolment into the study and continued through to the completion of acupuncture treatment. After a 2-month period of observation during which the participants recorded diary entries, they received 12 weekly acupuncture treatments, then 3 monthly treatments, for a total of 15 treatments over 6 months (Figure 1). Adverse events were recorded during each acupuncture session. Treatments were performed using standardized formula acupuncture and a set of 11 fixed acupoints: LR 3 *Taichong*, SP 6 *Sanyinjiao*, LI 4 *Hegu*, SP 8 *Diji*, SP10 *Xuehai*, PC 6 *Neiguan*, CV 6 *Qihai*, CV 3 *Zhongji*, ST 29 *Guilai*, BL32 *Ciliao*, Ex 22 *Zigong* (Table 1), bilaterally (except for Concetion Vassel points). These acupoints were selected because endometriosis is considered as stasis and deficiency of Blood and Qi in abdomen and in the uterus. Single effects of acupoints are described in Table 1.

**Table 1:** Acupoints in the treatment (formula acupuncture).

Acupoints	Insertion method	Effect
LR 3 *Taichong* (bilateral)	Insertion: perpendicular Depth: 0.5–1 cun *De Qi* tenderness and local distention, sometimes radiating to the fingers; numbness or mild electrical shock radiating to the sole	Treats stagnation of Qi of the liver. Promotes blood flow in *Chong Mai* and the uterus. Calms the mind. Analgesic action associated with LI4.
SP 6 *Sanyinjiao* (bilateral)	Insertion: perpendicular Depth: 1–2 cun *De Qi*: tenderness and local distention, sometimes radiating to the feet	Treats stagnation of blood, particularly in the lower *Jiao* and stagnation of *Qi* in the uterus. Nurtures *Yin* and the blood. Works against pathogenic factors such as Heat-Damp and Cold-Damp. Strengthens *Jing.*
LI 4 *Hegu* (bilateral)	Insertion: perpendicular Depth: 0.5–1 cun *De Qi*: numbness and tenderness radiating to the index finger	Relieves pain by unblocking circulation of *Qi* along the meridians (general analgesic-antispastic action)
SP 8 *Diji* (bilateral)	Insertion: perpendicular Depth: 0.5–1 cun *De Qi*: tenderness and local distention, sometimes radiating downwards	Treats stagnation of *Qi* and blood in the uterus. Regulates menstrual flow. Point *Xi* in lower abdominal pain (analgesic action in pain attacks).
SP 10 *Xuehai* (bilateral)	Insertion: perpendicular Depth: 0.8–1.5 cun *De Qi*: distention and local tenderness, sometimes radiating to the knee or the groin	Treats stagnation of the blood. Strengthens the spleen, nurtures, refreshes, and moves the blood. Acts on the uterus. Acts on irregular menses in association with SP8, especially in chronic disorders.
PC 6 *Neiguan* (bilateral)	Insertion: perpendicular Depth: 0.5–1 cun *De Qi*: numbness and/or mild electrical shock radiating to the hand, particularly to the middle finger	Treats stagnation of *Qi* of the liver. Calms the mind. Acts on the uterus.
CV 6 *Qihai*	Insertion: oblique downward Depth: 1.5–2 cun *De Qi*: numbness and tenderness radiating downward	Strengthens the *Qi* of the kidney and the entire organism and promotes circulation. Acts on the uterus. Treats Heat-Damp, Cold and stagnation of *Qi* in the lower *Jiao*.
CV 3 *Zhongji*	Insertion: perpendicular Depth: 0.5–1 cun *De Qi*: numbness and local tenderness radiating downward	Treats stagnation of *Qi* and Heat in the blood. Acts on the uterus. Strengthens *Jing* of the kidney. Eliminates Heat-Damp from the lower *Jiao*. *Mu* point of the bladder.
ST 29 *Guilai* (bilateral)	Insertion: perpendicular Depth: 0.5–1 cun *De Qi*: local numbness and tenderness	Treats stagnation of *Qi* and blood, Damp, Heat, and Cold in the lower *Jiao*, and the uterus in particular.
BL 32 *Ciliao* (bilateral)	Insertion: perpendicular Depth: 1–1.5 cun *De Qi*: distention and tenderness of the sacral region. Sometimes radiating to the lower limbs	Strengthens kidney and *Jing*. Treats Damp-Heat in the lower *Jiao*. Regulates urination and defecation.
EX 22 *Zigong* (bilateral)	Insertion: perpendicular Depth: 0.8–1.2 cun *De Qi*: local distention and tenderness	Strengthens *Jing* of the kidney. Acts on the uterus by regulating menstrual flow. Treats Heat-Damp in the lower *Jiao*.

Treatment sessions were performed in a quiet, temperature-controlled room and using sterile, single-use disposable fine steel needles (length 30 mm, diameter 0.3 mm). After insertion, each needle was manually manipulated to elicit De Qi sensation. The needles were then left in place without further manipulation for 30 min. All acupuncturists were physician acupuncturists with 4-year postgraduate training and at least 5 years of experience in acupuncture for gynecological disorders. Participant diaries kept during the observational period [baseline recording of two menstrual periods (T-2 and T-1)] were collected at the first session, and then again at session 4 (T1), session 8 (T2), session 12 (T3), session 13 (T4), session 14 (T5), and session 15 (T6). During the treatment period, the participants were allowed to continue their usual medications for symptom relief and were asked to record the frequency of use (always, nearly always, sometimes, rarely, never) and effect (ineffective, palliative, effective).

Statistical analysis was performed using Statistical Program for the Social Sciences (SPSS ver. 12). Continuous variables are expressed as means and percentile distribution; and categorical variable as percentages. The nonparametric Friedman test was used to investigate the overall significance of the differences between the mean data recorded at the time points of the study. Post hoc analysis was performed by multiple comparison with Bonferroni correction to assess significant differences in the data. Data on days of dysmenorrhea per month were analyzed using ANOVA for repeated measures followed by *post hoc* analysis with Bonferroni correction. To evaluate the degree of disability recorded at the time points during the study, two groups were formed: one with participants with moderate-to-severe disability, and the other with mild or no disability. The chi-square test was applied to determine differences before and after treatment. The significant level for any statistical analysis was defined as *p* value <0.03.

## Results

Table 2 presents the demographic and clinical characteristics of the 30 women who completed the study. Table 3 reported mean, median and percentile of clinical parameters evaluated during acupuncture treatment.

**Table 2:** Demographics and clinical characteristic of the study sample.

Demographics and clinical characteristics	Total Group (*n* = 30)
Age (years)	33.7 ± 7.4
Age at symptom onset (years)	17.8 ± 3.4
Age at diagnosis of endometriosis (years)	27.9 ± 5.9
Level of education (%)	
8 years	16.6
13 years	56.7
18 ± 3 years	26.7
Pregnancy (%)	
Nulliparity	63.3
One or more miscarriages or extrauterine pregnancy	16.7
One or more premature deliveries	13.3
One or more deliveries without complications	6.7
Number of previous surgeries (%)	
One	40.0
Two	33.3
Three or more	26.7
Classes of drugs used before enrollment (%)	
One	10.0
Two	36.7
Three or more	53.3

Plus-minus values are the mean ± standard deviation (SD).

**Table 3:** Mean, median and percentiles of clinical parameters evaluated in patients before (T-2) and at 13° session (T4).

	Mean	25° percentile	50° percentile	75° percentile
Dysmenorrhea intensity T-2	8.04	7.33	8.00	8.67
Dysmenorrhea intensity T4	3.41	3.00	3.5	4.00
Dysmenorrhea days T-2	2.90	2.50	3.00	3.00
Dysmenorrhea days T-4	1.28	1.00	1.00	2.00
Dyspareunia intensity T-2	5.74	4.00	5.00	6.00
Dyspareunia intensity T4	3.89	3.00	4.00	5.00
Non cyclic pelvic pain intensity T-2	6.28	5.00	6.00	7.00
Non Cyclic pelvic pain intensity T4	4.44	4.00	5.00	6.00
Dyschezia intensity T-2	4.15	3.00	3.50	5.00
Dyschezia intensity T4	3.05	2.00	3.00	4.00

**Figure 2:**
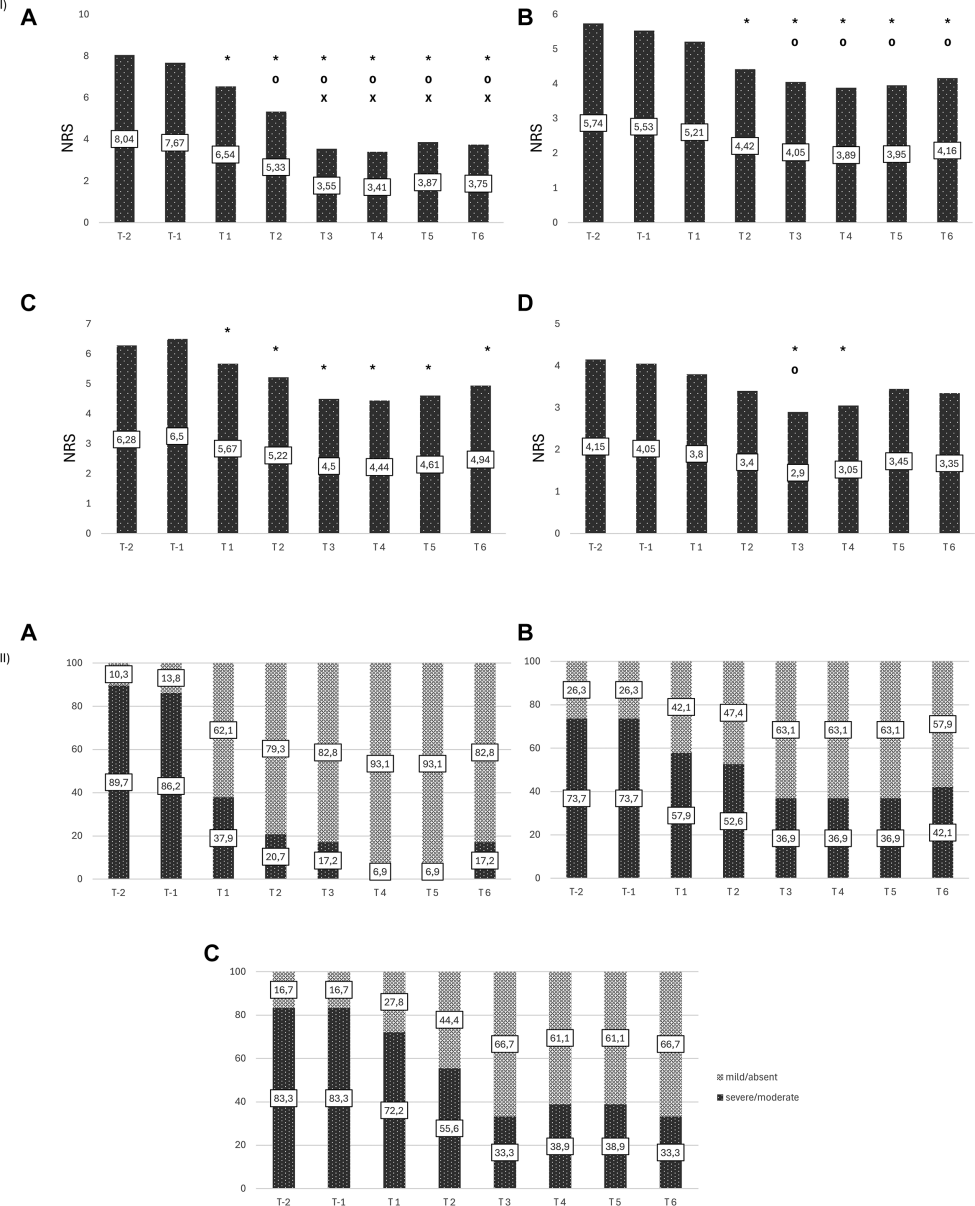
(**I**) Changes in intensity of endometriosis-related symptoms. (**A**) Dysmenorrhea (**B**) Dyspareunia (**C**) Noncyclical Pelvic Pain (**D**) Dyschezia. *Statistically significant vs. T_-2_ [(**A**) *p* < 0.0001; (**B**) *p* < 0.01; (**C**) *p* < 0.05; (**D**) *p* < 0.005)]; ⁰Statistically significant vs. T_1_ [(**A**) *p* < 0.0001; (**B**) *p* < 0.02; (**D**) *p* < 0.05)]; ^x^Statistically significant vs. T_2_ [(**A**) *p* < 0.0001)]. (II) Endometriosis-related disability. (**A**) Dysmenorrhea (**B**) Dyspareunia (**C**) Noncyclical Pelvic Pain.

**Figure 3:**
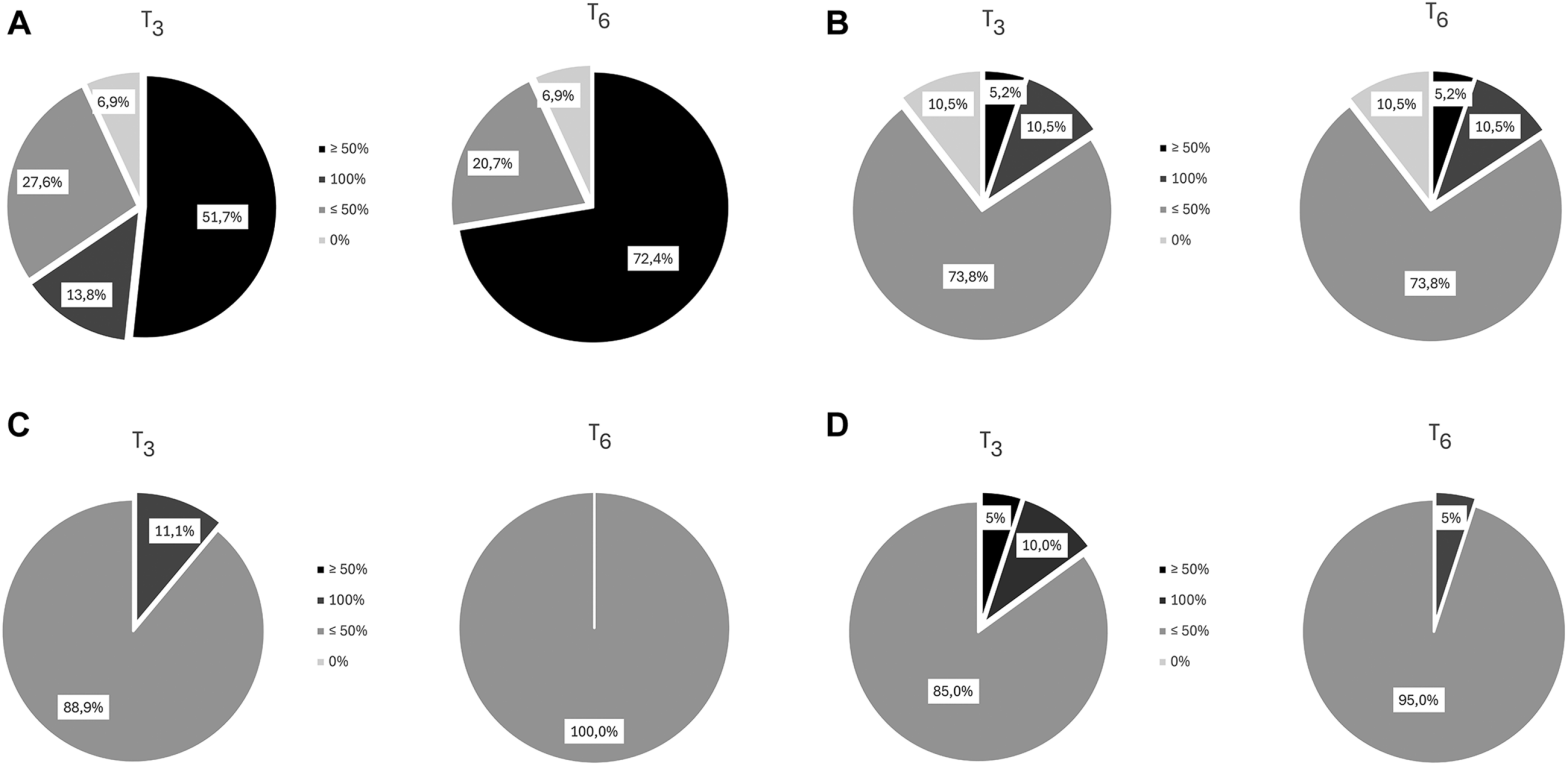
Percentage reduction in symptom intensity at T_3_ and T_6_. (**A**) Dysmenorrhea (**B**) Dyspareunia (**C**) Noncyclical Pelvic Pain (**D**) Dyschezia.

Four from the original sample of 34 dropped out because of personal reasons unrelated to treatment. Analysis of dysmenorrhea in 29 women (1 had undergone hysterectomy) showed a decrease from a mean intensity of 8.0 [(T-2) at baseline to 3.4 (T4), *p* < 0.001], followed by a slight increase over the next months (Figure 2IA); a decrease of at least 50% in dysmenorrhea intensity was noted in 65% at T3 and T6 (Figure 3A). The mean number of days of dysmenorrhea declined from 2.9 (T-2) to 1.28 (T4) and then remained stable over the next 2 months (*p* < 0.001). The number of days of dysmenorrhea decreased by at least 50% in 17 women (58.6%) at T3 and T6; freedom from symptoms was noted by 4 at T3, 3 at T4, and 2 at T5. The number of days of dysmenorrhea decreased from 4 to 3 days during the study period in the 2 women who experienced no improvement in pain symptoms.

The percentage of women with moderate-to-severe dysmenorrhea-related disability decreased from 89.7% at baseline (T-2) to 37.9% at T1, 17% at T3, and 6.9% at T4 and T5, whereas the percentage of those with no/mild dysmenorrhea-related disability increased from 10.3% at T-2 to 93.1% at T4 and T5. The number of women who reported no disability at the six time points increased during treatment (from 2 at T1, to 19 at T6); during the 2-month observation period, none of the women reported an absence of dysmenorrhea-related disability (pretreatment vs. treatment, chi-square, *p* < 0.001) (Figure 2IIA).

Nineteen women complained of dyspareunia, it was chronic in 8 (42.1%) and not chronic in the remaining 11 (57.9%). The intensity at baseline (T-2) was rated 5.7 and steadily decreased starting at (T2) to a minimum of 3.8 at month 4 of treatment (*p* < 0.0001) (Figure 2IB). Over 15% of the women reported a reduction of at least 50% in pain at T3 and T6 (Figure 3B). Fourteen (73.7%) reported moderate-to-severe dyspareunia-related disability at baseline (T-2); the percentage decreased to 57.9% at T1 and 36.9% at T3; it then remained unchanged over the 2-month observation period before rising to 42.1% at T6. The percentage of women with no or mild dyspareunia-related disability rose from 26.3% at baseline to 63.1% at months 3, 4 and 5 of treatment; 2 women reported no dyspareunia at three time points (T2, T3, T4); 1 woman reported no dyspareunia at T5 and T6 (pretreatment vs. treatment, chi-square, *p* < 0.02) (Figure 2IIB).

Eighteen women reported noncyclical pelvic pain rated 6.3 at T-2 and T-1. A statistically significant decrease in pain score was noted starting at month 1 of treatment in all women, with a mean score of 4.44 at T4 (*p* < 0.001) (Figure 2IC). The score remained unchanged over the next 2 months. A decrease of at least 50% was reported by 11.1% of the women at T3 (Figure 3C). Moderate-to-severe pelvic pain-related disability was reported by 83.3% at T-2 and T-1; the percentage of those with functional disability decreased to 33.3% at T3 and T6, when disability was rated as moderate. Starting at T3 5 women (27.7%) reported no disability (pretreatment vs. treatment, chi-square, *p* < 0.005) (Figure 2IIC).

Twenty women reported dyschezia, which was chronic in 8 (40%) and not chronic in the remaining 12 (60%). The intensity was rated 4.2 at baseline (T-2), decreased to 2.9 at T3, and then increased slightly. Two women reported no dyschezia at T3, and all reported a reduction in symptom intensity compared to baseline (Figure 2ID).

**Figure 4:**
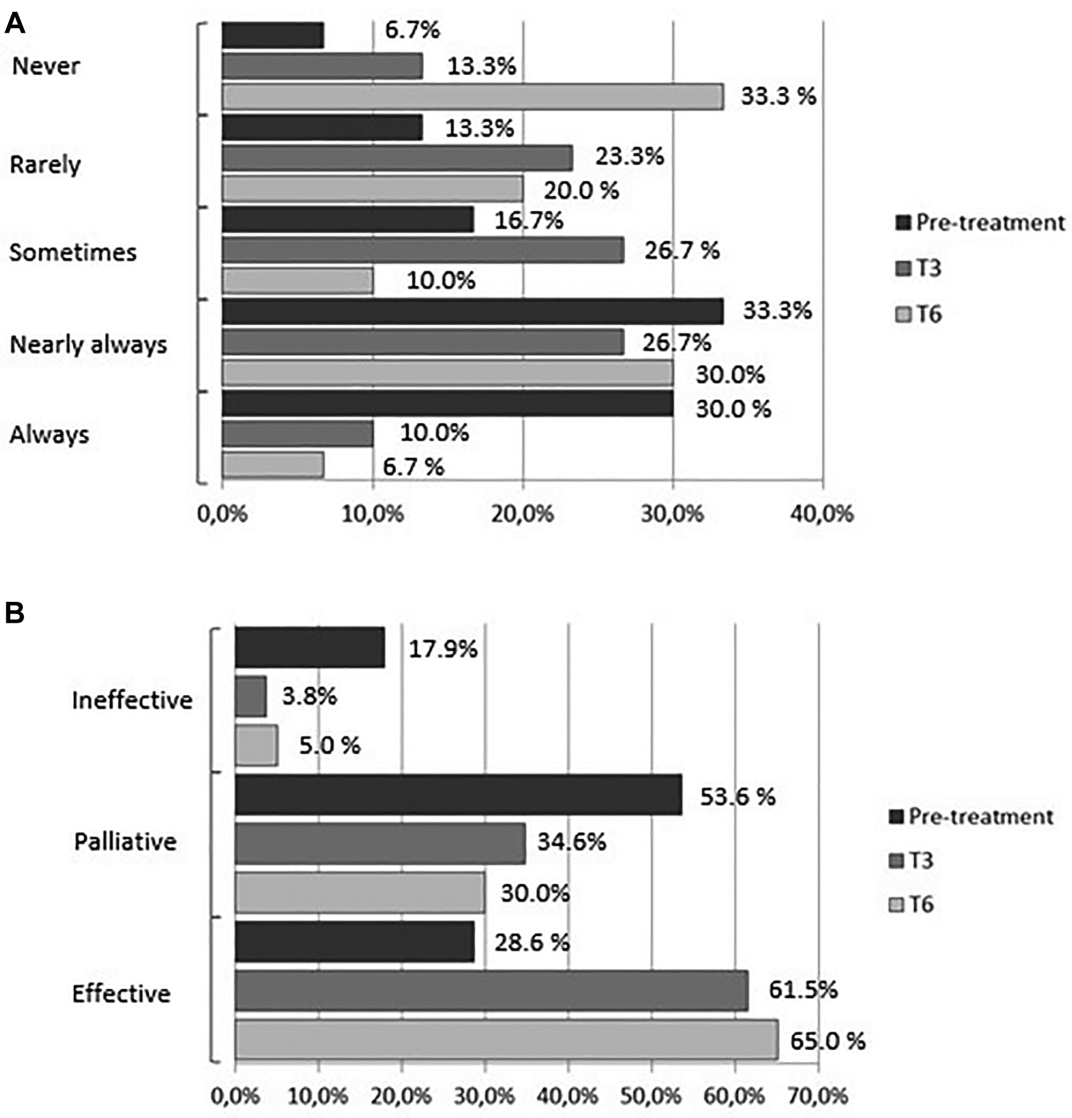
(**A**) frequency of medications use (analgesics/anti-inflammatory drugs). (**B**) Effect of medications (analgesics/anti-inflammatory drugs).

At baseline 63% of the women reported regular use of analgesics (NSAIDs) for acute pain relief (30% always, 33% nearly always): 30% reported occasional use of analgesics (17% sometimes, 13% rarely); 2 (7%) reported no use of medications; 29% reported relief of pain symptoms, 53% reported partial relief, and 18% reported no relief. The percentage of women who reported regular analgesics use (always) was 10% and 6.7% at T3 and T6, respectively; the percentage of those who used analgesics nearly always was 26.7% and 30%, respectively. At T3 half of the women reported using analgesics (23.3% rarely and 26.7% sometimes); at T6 the percentage dropped further to 30% (20% rarely and 10% sometimes), whereas the percentage of those who did not use medications rose from 13.3% at T3 to 33.3% at T6 (pre-treatment vs. treatment, chi-square, *p* < 0.05) (Figure 4A). Parallel to the decrease in the percentage of women who used analgesics regularly was an increase in the efficacy of treatment for symptoms: at 3 months of treatment, 61.5% of the women reported that medications were effective compared to 3.8% who reported no effect and 34.6% who reported a palliative effect. At T6, 65% of the women rated the effect of medications as effective compared to 30% and 5% who reported a palliative or no effect, respectively, (pretreatment vs. treatment, chi-square, *p* < 0.05) (Figure 4B).

## Discussion

Our findings suggest that acupuncture treatment is effective in reducing pain symptoms in women with deep endometriosis (dysmenorrhea, dyspareunia, pelvic pain, dyschezia). The greatest reduction was noted between month 3 and month 4 of treatment when reported symptom intensity was lowest: greatest reduction in dyschezia intensity (NRS score −1.2) or about 30% less at T3 compared to baseline (*p* ≤ 0.01); greatest reduction in dysmenorrhea intensity (NRS score −4.6) or over 50% less at T4 compared to baseline (*p* < 0.001); greatest reduction in dyspareunia (NRS score −1.84) or about 30% less at T4 compared to baseline (*p* = 0.002); greatest reduction in noncyclical pelvic pain (NRS score −1.83) or about 25% less at T4 compared to baseline (*p* = 0.005). A reduction in symptom intensity of at least 50% (range between 72% and 11% depending on symptom) for all symptoms except dyschezia compared to baseline was noted at the end of 15 acupuncture treatments.

Previous studies have reported a reduction of 33% (16) or 25% (17, 18) compared to baseline by month 3 of acupuncture or pharmacological treatment. Taking these values as reference points, we may state that a reduction in symptom intensity of at least 25% in the four symptoms was observed between month 3 and month 4 of acupuncture treatment in the present sample. A slight increase in symptom intensity was then noted, which was probably due to the switch from a weekly to a monthly treatment schedule. While the therapeutic response was maintained with single monthly sessions, the change in schedule was unable to confer further reduction in symptoms.

Analysis of secondary endpoints showed that acupuncture treatment played an important role in reducing the disability related to dysmenorrhea, dyspareunia, and noncyclical pelvic pain. Half of the women with moderate-to-severe dysmenorrhea-related disability at baseline reported an improvement at month 1 (89.7% at T-2 vs. 37.9% at T1) and only one-fifth reported no improvement at month 3 (89.7% at T-2 vs. 17.2% at T3). About half of the women reported a reduction in dyspareunia-related disability at month 3 (73.7% at T-2 vs. 36.9% at T3). This change was particularly meaningful for the women who underwent acupuncture treatment because of a desire to have children. A reduction of about 60% in moderate-to-severe disability related to noncyclical pelvic pain was observed at month 3 (83.3% at T-2 vs. 33.3% at T3). None of the women reported severe disability (albeit moderate) between month 3 and month 5 of treatment. At month 3 and month 6 of treatment, 65% of the women reported no dysmenorrhea-related disability and 27.7% reported no pelvic pain-related disability at month 3 of treatment.

A significant decrease in the number of days per menstrual cycle of dysmenorrhea was noted starting at month 2 of treatment, with the lowest number recorded at month 4 (−1.6) or a 50% reduction compared to baseline (*p* < 0.001); by months 3 and 6 a reduction ≥50% in the number of days of dysmenorrhea was reported by 58.6% of the women. Nearly half of the women reporting using analgesics (NSAIDs), whereas the percentage of those able to cope without analgesics increased five-fold. The percentage of responders to pharmacotherapy increased from 26.8% to 65% (baseline vs. T6).

Only 2 (6.7%) out of 30 women reported receiving no benefit from acupuncture treatment. Given the percentage of 20%–30% of non or low responders to acupuncture in the general population (19), our data indicate considerably higher than average response to treatment. No serious treatment-related side effects were reported, except for hematoma at the insertion site in 2 (6.7%). This rate is in line with previous studies that reported bleeding or hematoma at the needle insertion site, associated with pain and vegetative symptoms, which were not recorded in the present sample, however (20).

Our data indicate that acupuncture treatment was highly effective in reducing the intensity, duration, and functional disability related to dysmenorrheal. The reduction (albeit statistically significant) was less pronounced for symptom intensity related to dyspareunia and pelvic pain, although there has been a significant reduction in their related disability. An addition benefit of acupuncture treatment was the marked decrease in analgesics use during the study period. A plausible explanation for the significant reduction in dyschezia starting at months 3 and 4 of treatment is that monthly sessions may be too few to adequately manage this symptom.

Comparison of our data with previous studies investigating acupuncture for endometriosis is difficult because of the paucity of data (no studies on dyspareunia and dyschezia), the heterogeneity of study design and acupuncture formula, and patient selection. All the women in our sample had a diagnosis of DIE; published studies rarely indicate the stage of the condition. To date, only two studies have reported the disease stage: one reported on patients with stage II–IV endometriosis, without specifying how many patients at each stage (21); the other study stated that the patients had stage I–III disease but actually had stage I endometriosis (22).

Moreover, the extreme variety of acupuncture methods and protocols make it difficult to compare previous findings with ours. The one randomized controlled trial (RCT) on auricular acupuncture (23) cited in the 2011 Cochrane database systematic review (24) reported scores for pretreatment dysmenorrhea intensity as measured on a visual analogue scale (VAS) similar to the scores we observed in our sample, whereas the reduction at 3 months of treatment was greater compared to our data (−6.6 VAS vs. −4.5 NRS score). Another important difference is that the patients received daily acupuncture treatment over the 5 days before the first day of the menstrual cycle, which was far more frequent than according to our protocol.

Wayne et al. (22) reported slightly higher pretreatment VAS scores for pelvic pain than ours (7.4 VAS vs. 6.2 NRS score) and a greater reduction at weeks 4 and 8 than we found. Here, too, a plausible explanation for the difference is the twice-weekly schedule in treatment by Wayne et al. (once weekly in our study) and the lower stage of disease in Wayne et al.'s sample (stage I vs. stage IV).

Several mechanisms are involved in acupuncture effects. Acupuncture seems to alleviate pain by increasing pain thresholds in human and activating analgesic brain mechanisms through the release of neurohumoral factors (such as endogenous opioids) (25). Several studies have shown that acupuncture can modulated estrogen levels (26) and inflammatory pathways (27, 28). Down expression of peripheral blood CA 125 in patients treated with acupuncture would be a markers of remission or inflammatory modulation.

Our study has several limitations. The small sample size is consistent with the pilot nature of the study in which we wanted to test the treatment method and evaluate its efficacy in a small group before extending it to a larger scale. The small sample size is also due to the relative rarity and severity of the condition, as well as a result of strict patient selection criteria based on accurate disease staging. Another limitation is the lack of a placebo control group. In this setting it is extremely challenging to devise a valid placebo control method: so-called minimal acupuncture and the use of placebo devices with non-penetrating needles cannot be considered technically inert techniques (29–33).

A potential source of bias is that nearly half of the sample may be defined as “self-selected”: 16 women requested acupuncture treatment and 14 were referred by their gynecologist. This suggests that at least a part of the sample believed beforehand that they would benefit from acupuncture treatment; furthermore, belief in a treatment's efficacy is known to influence adherence to treatment and general therapeutic outcome (34, 35). Owing to the repeated failure of previous treatments, the women referred to our clinic by a specialist reported they did not have any particular expectations from acupuncture treatment. A previous study investigating the efficacy of acupuncture for endometriosis showed no marked differences in outcome in relation to patient expectations (22).

In conclusion, our findings cannot be generalized to all women with deep endometriosis; nonetheless, the study provides encouraging results. Future larger-scale studies may use to compare acupuncture and pharmacotherapy for endometriosis-related pain.

## Data Availability

The raw data supporting the conclusions of this article will be made available by the authors, without undue reservation.
